# Thermal Conductivity Measurement of Flexible Composite Phase-Change Materials Based on the Steady-State Method

**DOI:** 10.3390/mi13101582

**Published:** 2022-09-23

**Authors:** Ze Feng, Xin Xiao

**Affiliations:** 1 School of Environmental Science and Engineering, Donghua University, Shanghai 201620, China; 2 Yunnan Provincial Rural Energy Engineering Key Laboratory, Kunming 650550, China

**Keywords:** phase-change materials, enhancement, flexibility, thermal conductivity

## Abstract

Phase-change materials (PCMs) are widely used in energy storage and thermal management due to the large latent heat in the phase-change process. As one of the most significantly thermophysical properties of PCMs, the thermal conductivity has been extensively studied. Great attention has been paid to improving the thermal conductivities of PCMs; however, the studies on the thermal conductivities of flexible PCMs are relatively inadequate. In this study, polyethylene glycol 1500 (PEG1500) was used as the base PCM, and expanded graphite (EG) and styrene–butadiene–styrene (SBS) were added to improve the thermal conductivity and flexibility of pure PCMs, respectively. A steady-state experimental test rig was built and verified with the measurement of the thermal conductivity of stainless steel and deionized water, and then the thermal conductivities of PCMs at different phases and qualitative temperatures were measured extensively. Compared to the PEG1500 with 5 wt.% EG, the addition of SBS sharply reduces the thermal conductivity, which is only 0.362 W/(m·K) at 12.5 °C when the addition ratio is 50%. This is approximately a 69% reduction compared with the composite PCMs without SBS. Furthermore, the theoretical thermal conductivities of the composite PCMs were calculated with six theoretical models of multiphase systems. The majority of the models provide a good prediction of thermal conductivities of composite PCM with high SBS concentration, while the average deviation of Agari-Uno model is only 20.5% with different SBS concentration and relatively agrees well with the experimental results.

## 1. Introduction

Currently, massive fossil energy is consumed by automobiles with a large amount of waste gas exhaust, and this causes serious damage to the ecological environment. Therefore, as the preferred type of transportation for environmental protection and sustainable development, the demand and output of new energy vehicles have increased rapidly in recent years, along with lithium batteries. This is because lithium batteries are the power source of electric vehicles, and the loss rate is directly determined by the working temperature [1]. Jin et al. [2] indicated that the optimal working temperature of lithium battery was in the range of 20 to 40 °C, and the maximum local temperature difference was 5 °C. This could cause irreversible capacity loss to the battery if the working temperature exceeds the range or if the local temperature difference is overly large. In addition, there is a certain risk of heat accumulation at high temperature, inducing the battery burning or becoming thermally out of control [3,4]. Therefore, for the thermal management of a lithium battery, a method of passive temperature control of battery through phase-change materials (PCMs) to ensure the battery optimal lifespan has been proposed. Due to the poor heat dissipation of batteries by the PCMs of low thermal conductivity, the enhancement and accurate measurement of the thermal conductivity are becoming the current research hotspots. 

The traditional method is to integrate carbon materials with high thermal conductivities, such as expanded graphite (EG) [5], graphene [6], carbon nanotubes [7] and nano-metal particles, such as aluminum [8] and aluminum oxide [9], with pure PCM to significantly enhance the thermal conductivity. Zou et al. [10] used graphene and multi-walled carbon nanotubes (MWCNTs) to enhance the thermal conductivity of paraffin. The thermal conductivities of the paraffin/graphene and paraffin/MWCNTs composite PCMs were 0.75 and 0.61 W/(m·K), respectively, with an impregnation ratio of 1.5%, and these were measured using the transient plane heat source technique. Ling et al. [11] adsorbed sodium acetate trihydrate-urea (SAT-Urea) with EG, and the thermal conductivities of the composite PCMs increased from 0.61 to 4.19 W/(m·K) with the addition of 20 wt.% EG, which were also measured using the transient plane heat source technique. 

Krishna et al. [12] measured the thermal conductivities of tricosane-based composite PCMs by the hot-wire method, and the increase reached 32% by adding 2% Al_2_O_3_ nanoparticles. Mehrabi-Kermani et al. [13] impregnated paraffin into copper foam with the porosity of 0.903 and pore size of 20 PPI to make the heat dissipation faster of the lithium-ion battery, which was further enhanced by combining with forced-air convection caused by fans. The thermal conductivity of the composite PCM was 11.98 W/(m·K) measured by hot-wire method, which was 57 times than that of pure paraffin. 

Hussain et al. [6] attached a graphene coating to the surface of nickel foam, which had the porosity of 0.9 and pore size of 12.7 PPI and infiltrated with paraffin, improving the thermal conductivities of pure paraffin from 0.19 to 4.6 W/(m·K) measured by the laser flash method. Lin et al. [14] prepared a graphene skeleton by reducing graphene oxide and then impregnated paraffin into the porous structure under vacuum condition so that its axial and lateral thermal conductivities increased to 2.58 and 1.78 W/(m·K), respectively, which were also measured by laser flash method at room temperature. The increased ratios of the thermal conductivities reached 1260% and 840%, respectively. 

However, there are limitations in the measurements of thermal conductivities by transient plane heat source technique [11], hot wire method [13] and laser flash method [14]. Since these three methods do not distinguish the thermal contact resistance (TCR) of the sample surface from its own thermal resistance, the results obtained are the summation of both, indicating that the roughness of the sample surface would greatly affect the thermal conductivity [15]. 

Liu et al. [16] found that TCR played a dominant role in the total thermal resistance and was clearly influenced by changing the temperature, pressure and roughness of the contact. Ahadi et al. [17] analyzed the effective thermal resistance measured by conventional transient plane heat source technique and concluded that the proportion of TCR in total thermal resistance decreased with the increase in the thickness of sample, while the ignored TCR lead to the thermal conductivities having a 66.7% deviation from those measured with the steady-state method. As for the hot wire method, it was proposed to measure the samples with large size and smooth surface to reduce the uncertainty and the thermal conductivity of the measured samples is limited to 15 W/(m·K) [17,18]. The laser flash method is performed by heating one side of the sample with the pulse of energy, which results in the temperature on the back of sample rising to determine the thermal diffusivity. Due to this, TCR is regularly ignored, and the thickness of the sample is severely restricted by the timescales of the energy pulse and detection, and the specific heat must be known [19]. Xiao et al. [20] found that the uncertainty of thermal conductivity measured by the laser flash method can be as large as 40% when the TCR on both sides of the measured sample is ignored by numerical investigations. Additionally, these methods are not suitable for measuring the thermal diffusivity of thermal insulation materials [21]. 

In contrast, the steady-state method is more widely used with higher reliability. The heat flux and temperature gradient of the sample is kept stable in the steady state to obtain an accurate thermal resistance. For at least two samples with different thicknesses are measured, not only the effective thermal conductivities but the TCR values are obtained by the evolution of temperature in the direction of heat flux [22]. 

For instance, Xiao et al. [15] measured the thermal conductivities of copper foam with different porosities after paraffin adsorption using the steady-state method and found that the TCR accounts for 15%~50% of the total thermal resistance. Wang et al. [23] measured the thermal conductivities of the composite PCMs composed of paraffin, HDPE and EG with steady-state heat flux meter. The results showed that the thermal conductivities increased linearly with temperature in solid and liquid states, respectively, and the uncertainty was only 4.72%. Feng et al. [24] measured the thermal conductivity of paraffin by both transient plane heat source technique and steady-state method, and the experimental results showed that the thermal conductivity deviation between them could be less than 7% while the influence of TCR was considered. 

According to the differences of the shape, size, proportion, dispersion and interaction of fillers, different theoretical models were proposed to predict the theoretical thermal conductivities of the composite PCMs and then compared with the experimental results measured by above-mentioned methods [25,26,27]. In these models, the isotropic fillers with high thermal conductivity were considered as the dispersed phase and as dispersed in the polymer matrix, which was the continuous phase. Subsequently, several studies were reported for modifications, e.g., Wang et al. [28] proposed the influence of air with low thermal conductivity on porous matrix, and then Agari-Uno model was modified and effectively predicted the theoretical thermal conductivities of composite PCM, with a deviation of less than 9.6% from the experimental value. Xu et al. [29] introduced the contact resistance among fillers into the classical Maxwell model, and the predicted value by reconstruction of the Maxwell model agreed firmly well with the experimental results over a wide range of fillers concentrations and diameters. 

Another shortcoming for composite PCMs in practical applications is the insufficient flexibility, which restricts the reduction of the large TCR between PCM and battery and phase separation occurs during the recycling of PCM. In order to address the issues, the integration of copolymer with memory shape, such as styrene–butadiene–styrene (SBS), is proposed to have a good effect. By blending with the PCM to form a film on its surface, not only the flexibility is improved, but the PCM is prevented from separating out during circulation [30]. 

In the present study, polyethylene glycol (PEG1500) was used as PCM for the characteristics of being difficult to burn, while EG and SBS were used for synergistic packaging. A new type of flexible composite PCM was prepared. More importantly, a thermal conductivity determination system was built to accurately measure the thermal conductivity at different temperatures and additive mass fractions with steady-state method. In this way, the flexible PCM with suitable thermal conductivity was chosen, and its heat dissipation effects in the battery thermal management system are the main concern of future works. Additionally, the theoretical thermal conductivities of samples with different SBS concentrations were predicted by well-known semi-empirical models from the literature and compared with the experimental results.

## 2. Experimental Setup and Thermal Conductivity Measurement 

### 2.1. Preparation of PEG1500/EG/SBS Composite PCMs

In the present study, PEG1500 was used as the initial PCM, and EG was used to adsorb PEG1500 and improve the thermal conductivity of the composite PCM [31]. According to previous research [32], the adsorption effects of low EG content may not meet the requirement of preventing the leakage of the PCM together with the limited improvement of thermal conductivity, and it was observed that the shape of PCM is powdered with the low latent heat when EG content is overly large, which may be not suitable for battery thermal management. Thus, composite PCM with a suitable EG mass fraction was strongly proposed for battery thermal management [33]. Therefore, PEG1500/EG composite PCM was prepared with 95 wt.% PEG1500 and 5 wt.% EG as the matrix, and SBS was chosen to improve the flexibility and prevent leakage of PEG1500 by forming a film on the surface of EG [34]. The organic solvent dichloromethane (CH_2_Cl_2_) was used to dissolve SBS at room temperature due to its low toxicity and easy decomposition under light, which would not remain in the composite.

Firstly, after the proportion of SBS (Huizhou LCY Elastomers Co., Ltd., Huizhou, China) was determined, the SBS particles were dissolved into a gel-like solution by CH_2_Cl_2_ (Shanghai Gaoxinhuabo Instrument Co., Ltd., Shanghai, China) and the PEG1500 (Sinopharm Chemical Reagent Co., Ltd., Shanghai, China) particles were placed in water bath at a temperature of 70 °C until it was melted. EG (Nantong Yifan Graphite Co., Ltd., Nantong, China) was prepared by placing expandable graphite in a muffle furnace at 850 °C for about 10 mins, which was then added into the molten PEG1500. Secondly, the above-mentioned composite was stirred with a glass rod for about 15 mins until it was fully mixed and solidified at room temperature, which was subsequently crushed and ground into powder. Then, the PEG1500/EG powder was added into the solution of CH_2_Cl_2_ containing SBS and stirred for about 15 mins to become effectively wrapped by the SBS, whose contents were 10 wt.%, 20 wt.%, 30 wt.%, 40 wt.% and 50 wt.%, respectively. Finally, the flexible composite PCM powder was placed in a ventilated place for 4 hours to completely volatilize CH_2_Cl_2_ and then pressed into PCM blocks with the diameter of 50 mm and thicknesses of about 15, 30 and 45 mm, respectively, under a constant pressure of 6 MPa by a pelletizer (769YP-15A, Tianjin Keqigaoxin Technology Co., Ltd., Tianjin, China). The SBS content and the detailed values of these samples are listed in Table 1, and the addition of SBS was used to improve the flexibility of materials. 

### 2.2. Experimental Device

**Figure 1:**
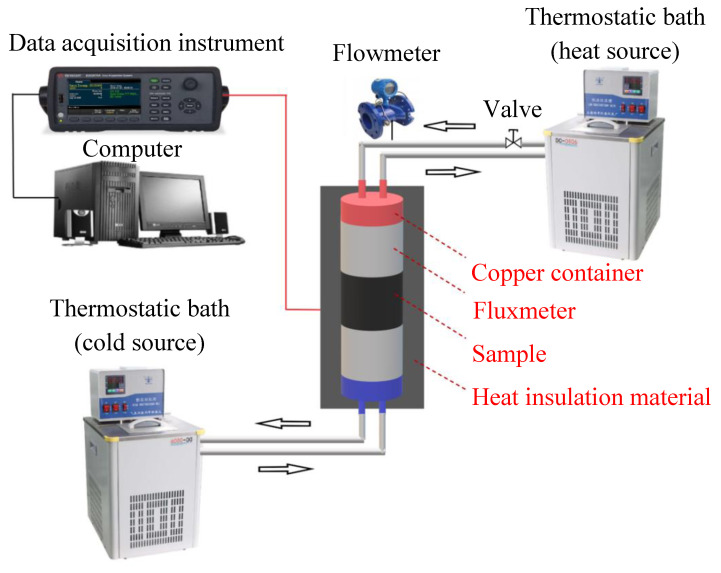
Schematic diagram of the experimental system.

In the present study, a steady-state system with constant heat sources was used to test the thermal conductivities of cylindrical samples with different thicknesses. The structural diagram of the experimental system is shown in Figure 1. The upper constant heat source was controlled by the water circulation of the thermostatic bath (DC2010, Shanghai Hengping Instrument Co., Ltd., Shanghai, China), and the lower cold source at the bottom was provided by the ethylene glycol/water mixture (i.e., 1:3) circulating in another thermostatic bath (DC2010, Henan Xinling Instrument Co., Ltd., Henan, China). 

For the power difference of circulating pump in thermostatic bath, a valve with an electronic flowmeter was installed between the heat source and thermostatic bath to control the flow rate, which was set to 0.7 L/min during the experiment. The heat flow was conducted from the heat source to the cold source through upper fluxmeter, samples and lower fluxmeter then adjusted to be stable after it reached a steady state.

**Figure 2:**
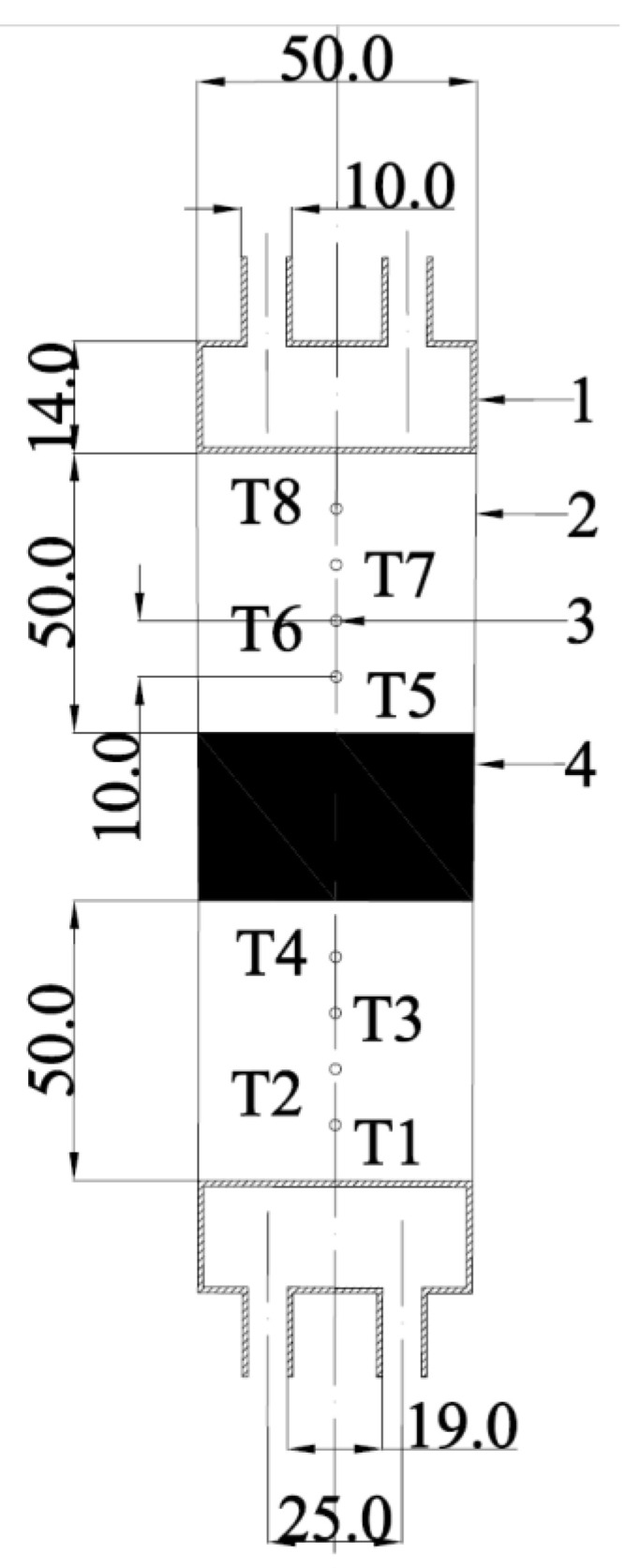
Schematic diagram and parameters of the test section. (1) Copper container, (2) fluxmeter, (3) platinum resistance thermometer and (4) sample to be tested (unit: mm).

Figure 2 shows the detailed parameters of the test section and its schematic diagram. The test section consists of two copper containers with diameters of 50 mm as heat and cold sources, upper and lower fluxmeters as the heat transfer media and the temperature measurement section, where the fluxmeters made of 304 stainless steel are with the diameter of 50 mm. Each fluxmeter was perforated with four holes with a diameter of 2 mm up to the axis as shown in Figure 2. 

In this way, eight platinum resistance thermometers of PT100 (i.e., T1–T8) coated by thermal conductive silicone grease were placed into holes to obtain the temperature of the fluxmeters with a data acquisition instrument (DAQ970A, Keysight Technology Co., Ltd., Penang, Malaysia) for subsequent thermal conductivity calculations. 

**Figure 3:**
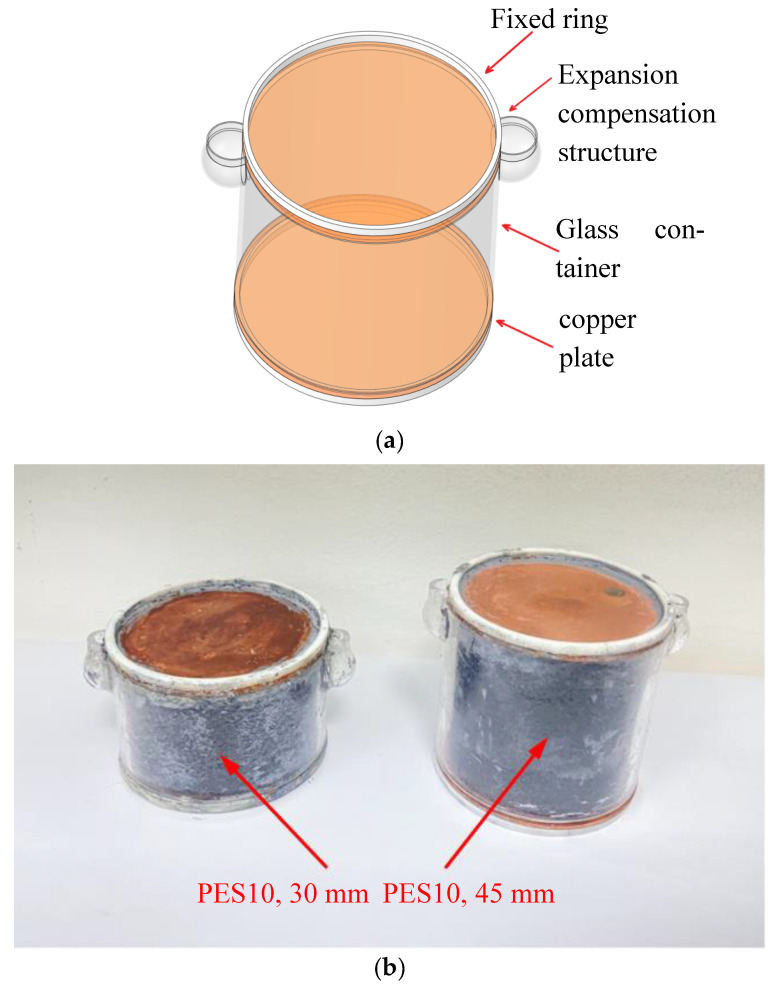
Schematic diagram of the sample containers in the liquid state. (**a**) Glass container and (**b**) layout of samples.

During the experiment, the upper and lower thermostatic baths were set to different temperatures to measure the thermal conductivities of solid samples at different temperatures. As for the liquid or molten samples at high temperature, a custom-made glass container was proposed as shown in Figure 3. The container body was made of glass with the upper and lower parts sealed by copper plates of the thickness of 2 mm, and two nylon rubber rings with the thickness of 2 mm were attached to the edge of the copper plate to fix the container, which was done to ensure that the fluxmeter was consistent with the sample in the vertical direction and the heat flux flowed vertically. 

In addition, the container was also designed with two expansion compensation structures due to the volume change when the phase of PCM changes. The liquid in the compensation structures flowed back into the container to ensure that the solution was in close contact with the top copper plate without any gap when the volume of the solution decreased. While the volume of the solution expanded, the liquid was squeezed to compensate the rise of the liquid level in the structure, keeping the pressure balanced in the container. 

**Figure 4:**
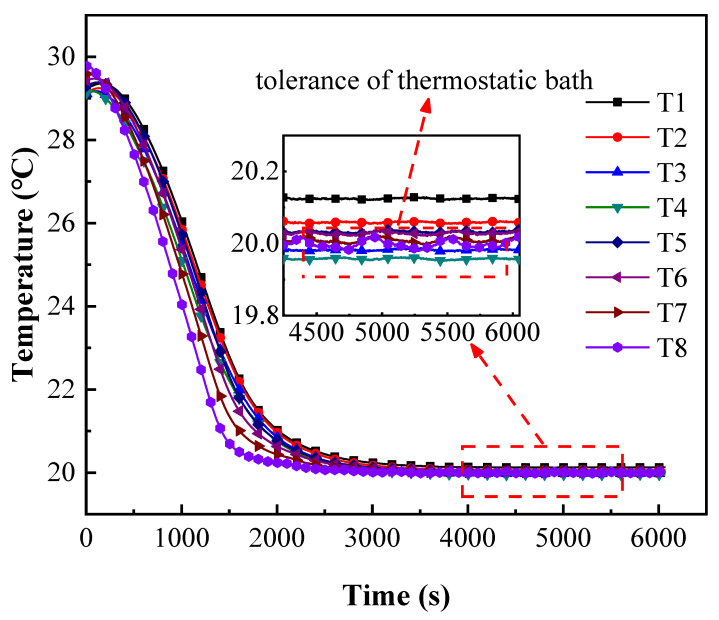
Pre-calibration of the platinum resistance thermometers at 20 °C.

It should be noted that the contact surfaces of copper pieces, fluxmeters and samples were coated with thermal conductive silicone grease to decrease the TCR ratio regarding the total thermal resistance. The experimental device was tightly coated with several layers of thermal insulation materials, which is silica aerogel pad with a thermal conductivity of 0.019 W/(m·K) and a rubber–plastic sponge with a thermal conductivity of 0.034 W/(m·K), to prevent the influence of external temperature on the heat flow, while the heat leakages through the platinum resistance thermometers were estimated to be 1.16% compared to the axial heat flux. The temperature evolutions of platinum resistance thermometers were verified to be less than 0.1 °C when both thermostatic baths were set as 20 °C as shown in Figure 4. As of the tolerance of the thermostatic bath is the cold source, the fluctuations of temperatures of T8 and T7 were within 0.01 °C, which can be ignored in the experiments.

During the experiment, the heat flow from the upper copper container reached the lower copper container through fluxmeters and samples. Since the temperatures of the cold and heat sources were constant, the system quickly reached a steady state within 60 mins and then was maintained for 30 mins to obtain the averaged temperatures of T1–T8, which were obtained by connecting platinum resistance thermometers with a data acquisition instrument. 

### 2.3. Experimental Method

Since the diameter of fluxmeters are the same as those of the samples to be measured with a uniform wall temperature provided by cold and heat sources and the thermal insulation measures are taken in the radial direction, the whole process is considered as an axial one-dimensional heat transfer process. The heat flux of each interface is almost the same and can be given by Fourier’s Law (1):

(1) $$q=-\lambda_{st}\times \frac{dt}{d\delta }$$

(2) $$\lambda_{st}=11.05+0.0525\times T_{qua}$$

where *λ_st_* is the thermal conductivity of the stainless steel fluxmeter, and it can be seen from Equation (2) that the temperature deviation *dt* is measured by the platinum resistance during the experiment. The distance *dδ* is determined by the test rig. *T_qua_* in Equation (2) indicates the qualitative temperature of stainless steel fluxmeter at the specified position, which is generally averaged from the temperatures of adjacent measuring points.

The temperature difference between the top and bottom of a sample can be deduced from the temperature distribution of the fluxmeter. The average heat flux of the fluxmeter is approximately regarded as that passing through the sample. As a result, the total thermal resistance of the samples to be measured is deduced by Equation (3).

(3) $$R_{all}=\frac{\Delta T_{s}}{\bar{q}}=TCR+R_{s}=TCR+\frac{\delta }{\lambda }$$

where *R_all_* is the total thermal resistance of the sample, including the thermal resistance *R_s_*, and the TCR, *δ* and *λ* are the thickness and the thermal conductivity of the sample respectively. ∆*T_s_* is the temperature difference between the upper and lower interfaces of the sample, and $\bar{q}$ is the average heat flux of the fluxmeter.

The total thermal resistance can be obtained by the experiments, while TCR is considered to be a constant for same types of samples as the surface of the sample is covered with thermal conductive silicone grease. Therefore, the thermal conductivity of the sample can be determined by the total thermal resistance at different thicknesses, and to further reduce the tolerance of the results, the total thermal resistance of each thickness is obtained by averaging three independent experimental results, where the properties of the samples are listed in Table 1.

### 2.4. Experimental Uncertainty Analysis

During the measurement of thermal conductivity in the present study, the uncertainty analysis of related parameters is involved in Equation (4):

(4) $$R_{s}=f\left(q,\;\Delta T,\;h,\;A\right)$$

The total uncertainty of thermal resistance is obtained by the tolerance propagation law, which depends on the uncertainty of heat flux Δ*q*, temperature difference Δ*t*, thickness difference Δ*h* and the contact area difference Δ*A*. The typical sample PES0 with the thickness of 45.84 mm under the condition of heat and cold sources temperature of 35 and −10 °C, respectively, is taken as an example, and the uncertainty of each parameter with detailed calculation processes are shown in Table 2. The tolerances of testing instruments are considered to be the reason for experimental uncertainty, such as the platinum resistance thermometer, data acquisition instrument, vernier caliper and thermostatic bath. In addition, the testing section can not be completely heat insulated, leading part of heat flow transferred from the radial direction to the outside, which changes the radial temperature distribution.

## 3. Experimental Results and Analysis

### 3.1. Verification of Experimental System

The verification of thermal conductivity measurements by the system includes the experiments of the solid and liquid samples. The former was verified by measuring the thermal conductivities of 304 stainless steel at the temperatures of 12.5, 22.5, 32.5, 42.5, and 52.5 °C, in comparison with Ref. [15], while the latter was verified by measuring the thermal conductivities of deionized water at the temperatures of 20 and 40 °C, in comparison with Ref. [35].

**Figure 5:**
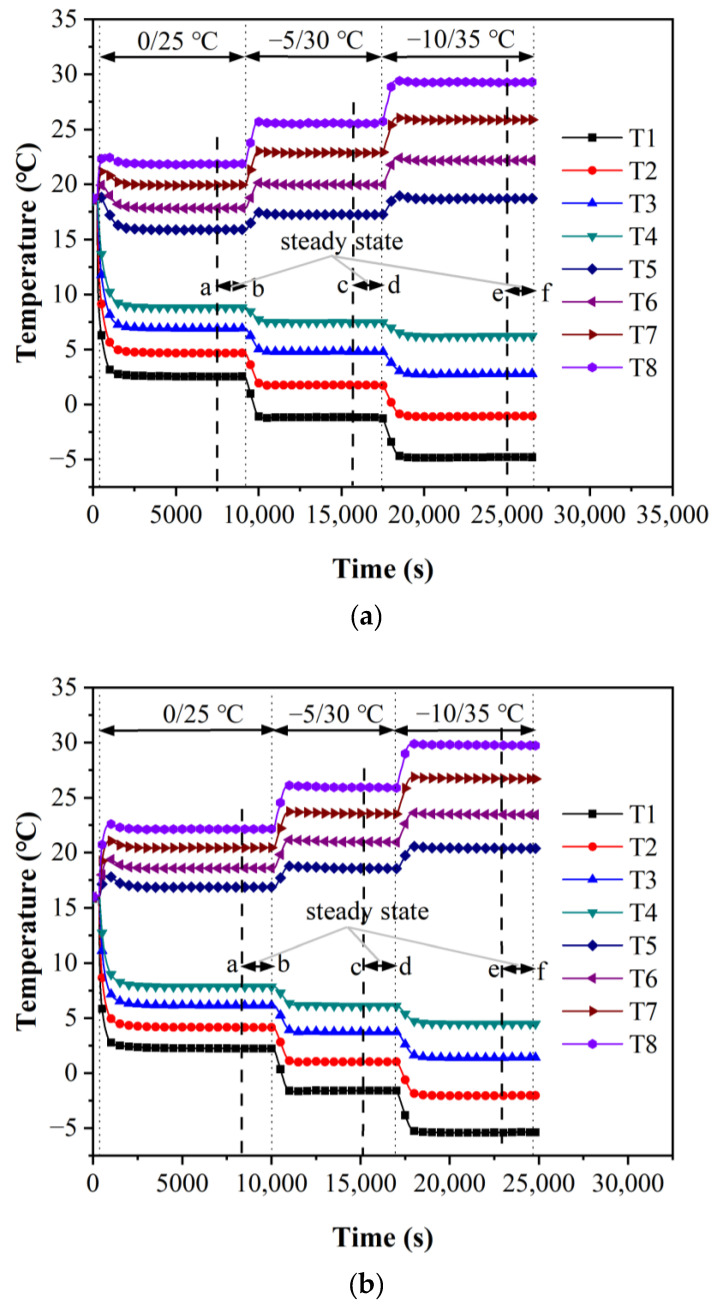

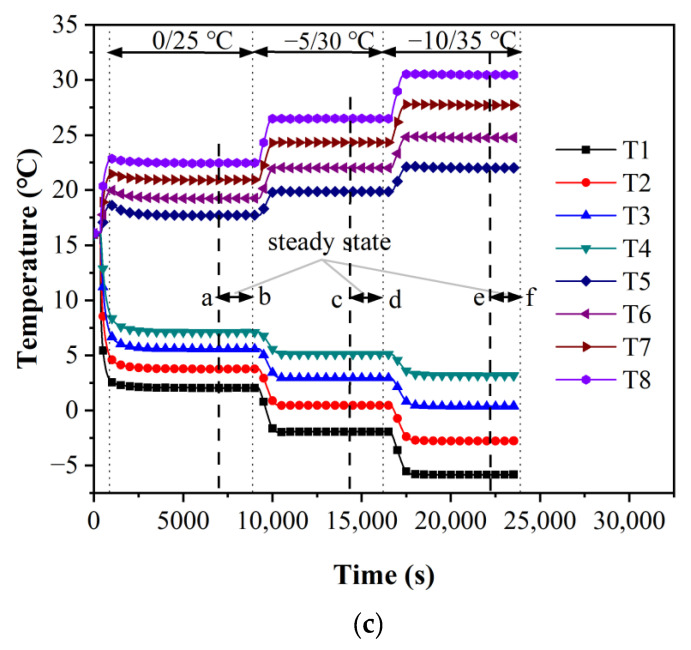
Experimental results of 304 stainless steel with the qualitative temperature of 12.5 °C. (**a**) *h* = 10.22 mm, (**b**) *h* = 25.06 mm and (**c**) *h* = 40.02 mm.

The temperature variations of stainless steel during the experiment are shown in Figure 5, where the qualitative temperature is 12.5 °C as the example. After the temperature of the heat and cold sources is set as 25/0 °C, the circulating water temperature in the thermostatic bath begins to change rapidly, leading the heat flow to transfer from top to bottom until reaching a steady state after about 5000 s as shown in sections ab in Figure 5, which is kept for approximately 2000 s to estimate the actual thermal resistance through the stable temperature gradient. 

**Figure 6:**
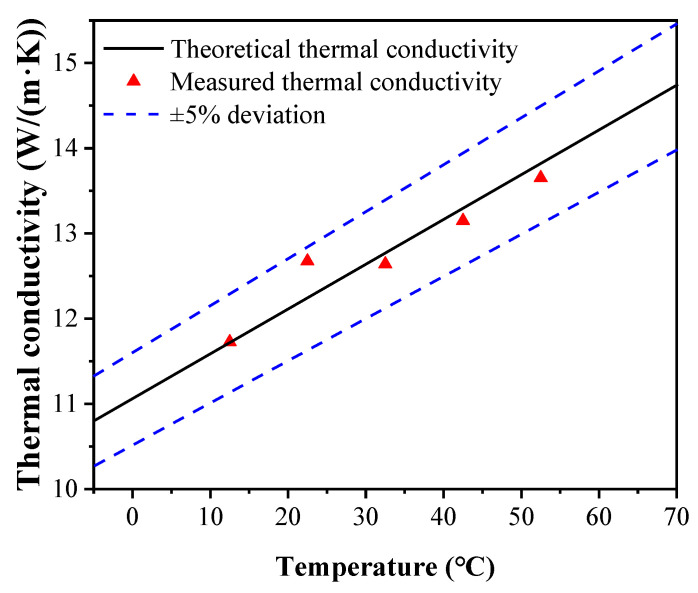
Comparisons between the experimental results and literature data [15] of 304 stainless steel.

Subsequently, the temperature of the thermostatic baths is set as 30/−5 and 35/−10 °C, respectively, to obtain the thermal resistance in the other two steady states represented by sections cd and ef in Figure 5. During the whole process, the other two sets of data are measured for stainless steel samples with the qualitative temperature at 12.5 °C to eliminate the tolerances and by changing the temperatures of heat and cold sources. Those of stainless samples at different qualitative temperatures are obtained by the experimental test rig, and the experimental results are shown in Figure 6, including the values of Ref. [15]. As the maximum deviation is 3.6%, the experimental results agree reasonably well with those of the reference, indicating the high efficiency with reliability of the experimental system.

**Figure 7:**
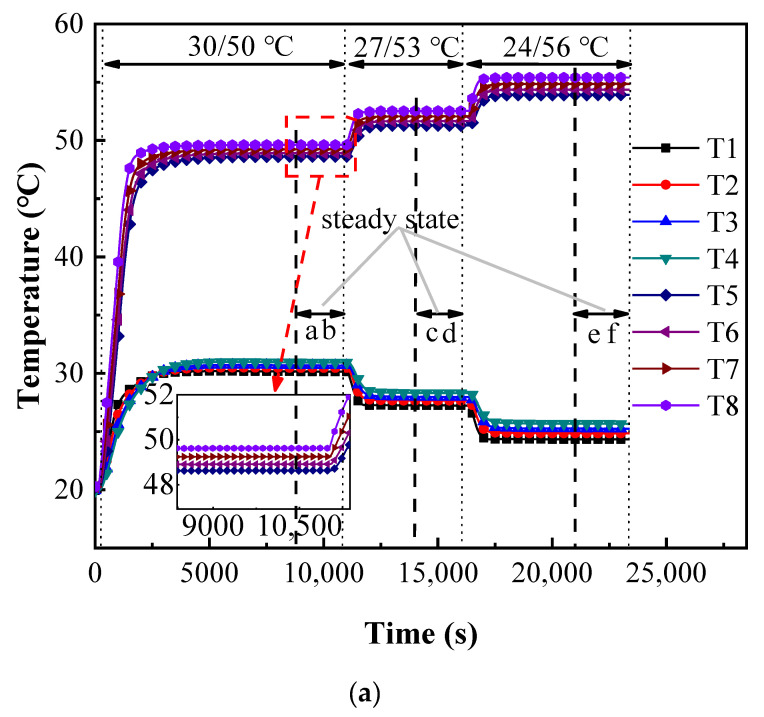

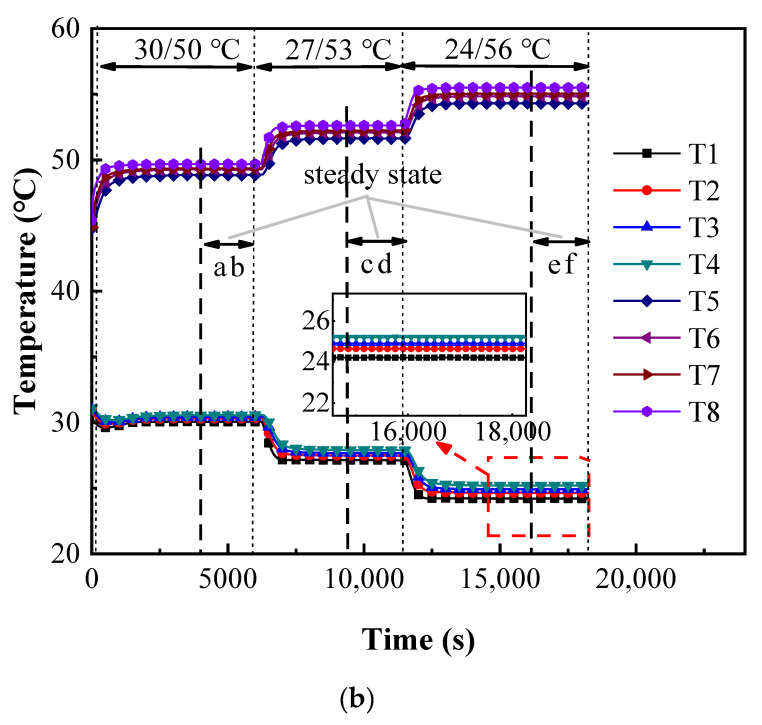
The experimental results of deionized water with the qualitative temperature of 40 °C as the example. (**a**) *h* = 30 mm and (**b**) *h* = 45 mm.

As for the liquid samples, due to the inner diameter of the glass container being larger than the fluxmeter, the heat from heat source is almost conducted through specimens to the cold source and little influence is caused by the axial heat flux transfer. However, it should be noted that the main experimental uncertainty is from the expansion compensation structure of glass container. It changes the cylindrical shape of the liquid samples and causes local heat loss. Therefore, it is designed to be small enough to reduce the experimental deviation. The temperature curves of deionized water are shown in Figure 7, where the qualitative temperature is 40 °C. Similar to the experimental results of stainless steel, the steady-state stage can be quickly obtained after the temperatures of cold and heat sources are set, which shows the high efficiency of the experimental system designed in the present study. 

As shown in the enlarged diagram in Figure 7, the temperatures of the fluxmeters are firmly stable in the steady state as well as the interval from T1 to T8, indicating the uniform distribution of heat flow. However, it is clear that the temperature evolution between these platinum resistance thermometers in the steady-state stages of water is greatly reduced. Although the temperature difference between the hot and cold sources reaches 32 °C, that of two measured points is about 0.3 °C, which is far less than the 2.5 °C of the stainless steel samples. 

It is consistent with the inference of Fourier law that the thermal conductivities of the water are much lower than those of stainless steel with the larger thermal resistance and reduces the heat transfer from the upper fluxmeter to the lower one to lead a smaller temperature gradient. Interestingly, the temperature difference between the upper and lower fluxmeters is also apparent and causes a slight deviation of the thermal resistance. Therefore, the axial heat flux is averaged to calculate the effective thermal conductivity. The thermal conductivities of deionized water at temperatures of 20 and 40 °C are 0.653 and 0.689 W/(m·K), while those in Ref [35] are 0.599 and 0.635 W/(m·K), respectively. The deviations of 8.3% and 7.8% indicate that the method is reliable for measuring liquid samples.

### 3.2. Effective Thermal Conductivity of Flexible Composite PCM

The thermal conductivity of pure SBS was measured first. The total thermal resistance of SBS blocks with thicknesses of 12.89, 18.77 and 26.03 mm were tested and linear fitted, indicating that the thermal conductivities of pure SBS were 0.182 and 0.188 W/(m·K) at the temperatures of 12.5 and 30 °C, respectively, which increased 3.3% with the higher temperature. Although the thermal conductivities of SBS have not been measured in the previous study, it was considered to be close to those of paraffin (0.23 W/(m·K)) [30], which showed agreement with the present results. Furthermore, those of pure PEG1500 were measured to be 0.346 and 0.353 W/(m·K) at 12.5 and 27 °C respectively, which agreed well with the value of 0.31 W/(m·K) in Ref. [36].

**Figure 8:**
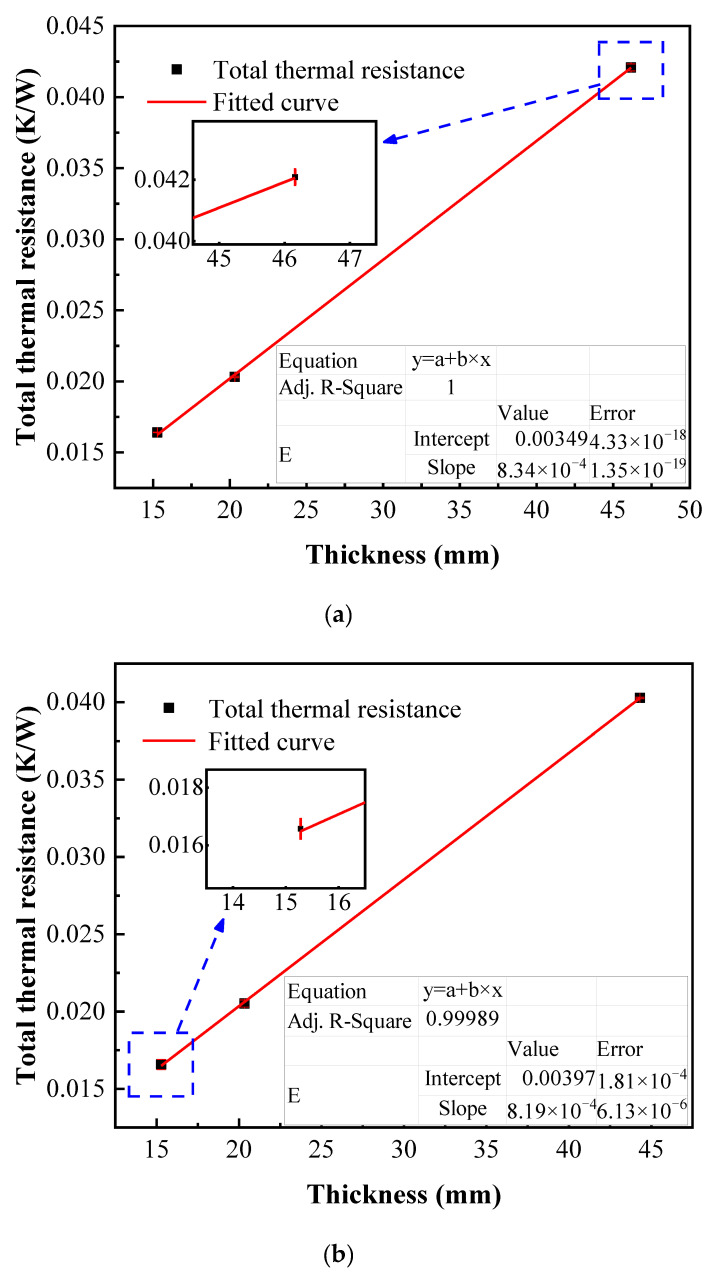
The total thermal resistances of SEP10 with different thickness. (**a**) Qualitative temperature of 12.5 °C and (**b**) qualitative temperature of 27 °C.

Each thermal conductivity is obtained by the linear dependence of the total thermal resistance on three different thicknesses. We mentioned above that the total thermal resistance is measured at three steady states to obtain the average value and the experimental deviation is extremely small as shown in Figure 8. Considering PES 10, the maximum deviation of total thermal resistance is only 4.1% with the average deviation of 1.37%, which indicates the reliability of the experimental system in the present study.

**Figure 9:**
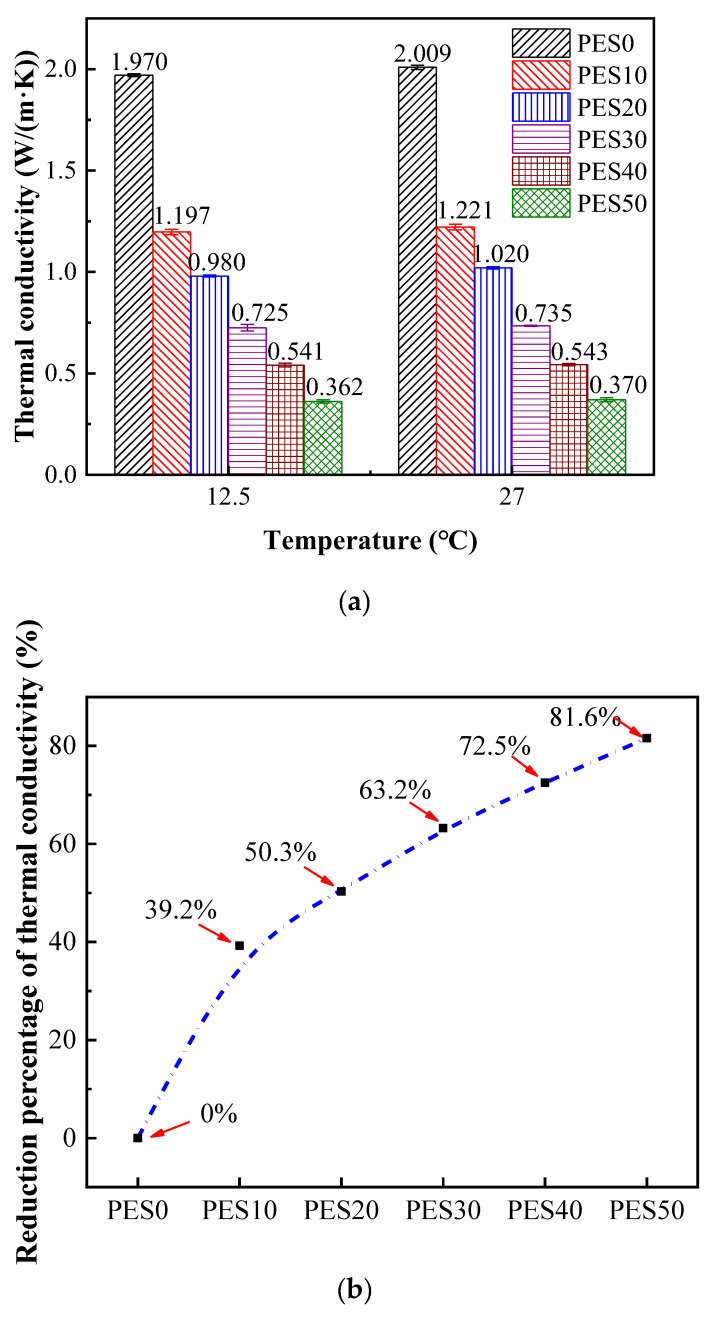
The thermal conductivities and reduction percentages of solid samples. (**a**) Samples of different SBS concentrations at 12.5 and 27 °C. The error bars are the standard deviation of the measurements (n = 3). (**b**) the reduction percentages of thermal conductivities compared to PES0 at 12.5 °C.

Figure 9a shows the experimental results of the thermal conductivities of the solid flexible composite PCMs at different temperatures. Then, the experimental results at 12.5 °C are discussed as an example. First, the thermal conductivity of PEG1500/EG is as large as 1.970 W/(m·K), while that of pure PEG1500 is 0.346 W/(m·K) [36] due to the effective paths for heat conduction formed by EG particles. After being integrated with 10 wt.% SBS, the value drops sharply to 1.197 W/(m·K), and the other values are 0.980, 0.725, 0.541 and 0.362 W/(m·K), when the addition ratios of SBS are 20 wt.%, 30 wt.%, 40 wt.% and 50 wt.%, respectively. 

Those results are the average values of different heat flows at the same qualitative temperature. Additionally, the error bars show the high repeatability of the experiments, considering the maximum deviation of thermal conductivities is less than 4.3%. As shown in the Figure 9b, the initial addition of 10 wt.% SBS reduces the thermal conductivity of PCM by 39.2% and the latter 10 wt.% SBS further reduces it by about 10%, and the slope of the curve noticeably decreases. 

We conclude that the excellent paths for heat conduction of EG are destroyed when its surface is covered with a film of SBS, which restricts the transfer of heat. In this way, although the thermal conductivity of composite PCM keeps decreasing with the increase in the SBS content, the decreasing range is not as apparent as the surface of EG is almost covered by SBS. 

**Figure 10:**
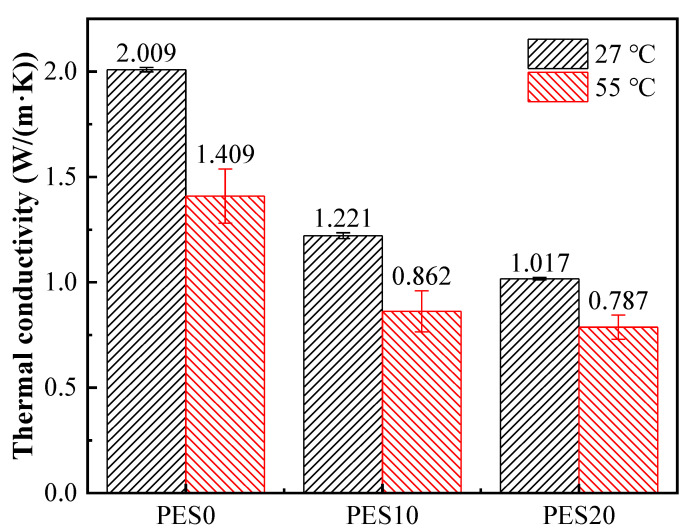
The thermal conductivities of samples with different SBS concentrations at 27 and 55 °C. The error bars are the standard deviation of the measurements (n = 3).

Since the slow accumulation of heat in the process of battery thermal management may be caused due to the low thermal conductivity of PCMs, the PES 20 with thermal conductivity of 0.980 W/(m·K) is taken as the acceptable one of the flexible PCMs. In addition, the trend of the thermal conductivities with the temperature is raised to be the same. When the temperature varies from 12.5 to 27 °C, the thermal conductivities of different samples increase accordingly, although this is limited. It should be noted that the increased percentages of PES0 to PES50 are 2.0%, 2.0%, 4.1%, 1.4%, 0.4% and 2.8%, which are irregular with respect to different SBS concentrations. The possible reason is that the increases in the thermal conductivity of SBS and PES0 with temperature are similar, 3.3% and 2.0%, respectively. However, when the temperature is higher than the phase-change temperature of the composite flexible PCMs, the thermal conductivities in the present study decrease in relatively large percentages as shown in Figure 10. 

The thermal conductivities of the flexible materials PES10 and PES20 and the substrate PES0, which have high thermal conductivities, and the prospect of battery thermal management are measured at high temperature. The thermal conductivities of PES0, PES10 and PES20 decrease from 2.009, 1.221 and 1.017 W/(m·K) to 1.409, 0.862 and 0.787 W/(m·K), respectively, reducing by approximately 29.9%, 29.4% and 22.6%. 

This may be due to the inherent low thermal conductivity of the molten PEG1500; however, the addition of SBS appears to reduce the declining trend of the thermal conductivities. It can be predicted that the encapsulation effect of SBS with stable thermal conductivities reduces the leakage of PEG1500 impregnated with EG. Due to this, the heat accumulation at high temperatures in battery thermal management using the flexible PCMs can be reduced in an effective way.

### 3.3. Theoretical Prediction of Thermal Conductivity

In the present study, the thermal conductivities of the composite flexible PCMs at 27 °C were theoretically predicted. During the preparation process of the composite PCMs, different raw materials were physically mixed, indicating that the dispersed phase particle EG was uniformly dispersed in the PCM, and thus it was considered as a composite material with dispersed high thermal conductivity fillers in the continuous polymer matrix. Based on factors, such as the shape, size, proportion, dispersion and interaction of fillers, its effective thermal conductivities were predicted by well-known theoretical models for multiphase composite as shown in Table 3, and the rationality and theoretical reasons for its change in EG content were verified by comparison with the experimental results.

**Table 3:** Predictions for the effective thermal conductivity of the multiphase composite using theoretical models from the literature.

Items	Theoretical Predictions	Equations
Russell model [25]	$\lambda_{eff}=\lambda_{m}\frac{\phi^{\frac{2}{3}}+\frac{\lambda_{m}}{\lambda_{f}}(1-\phi^{\frac{2}{3}})}{\phi^{\frac{2}{3}}-\phi +\frac{\lambda_{m}}{\lambda_{f}}(1+\phi -\phi^{\frac{2}{3}})}$	(5)
Cheng-Vachon model [26]	$\frac{1}{\lambda_{eff}}=\frac{1-A}{\lambda_{m}}+\frac{\ln\frac{{\left\{\lambda_{m}+A(\lambda_{f}-\lambda_{m})\right\}}^{\frac{1}{2}}+\frac{A}{2}{\left\{B(\lambda_{f}-\lambda_{m})\right\}}^{\frac{1}{2}}}{{\left\{\lambda_{m}+A(\lambda_{f}-\lambda_{m})\right\}}^{\frac{1}{2}}-\frac{A}{2}{\left\{B(\lambda_{f}-\lambda_{m})\right\}}^{\frac{1}{2}}}}{{\left[B(\lambda_{f}-\lambda_{m})\{\lambda_{m}+A(\lambda_{f}-\lambda_{m})\}\right]}^{\frac{1}{2}}}A={\left(\frac{3\phi }{2}\right)}^{\frac{1}{2}}B={\left(\frac{2}{3\phi }\right)}^{\frac{1}{2}}$	(6)
Agari-Uno model [27]	$\lambda_{eff}=(\frac{\lambda_{f}}{C_{p}\lambda_{m}}{)}^{\phi C_{f}}\times C_{p}\lambda_{m}$	(7)
Meredith-Tobias model [37]	$\lambda_{eff}=\lambda_{m}\left[\frac{2(2+\frac{\lambda_{f}}{\lambda_{m}})-2\phi (1-\frac{\lambda_{f}}{\lambda_{m}})}{2(2+\frac{\lambda_{f}}{\lambda_{m}})+\phi (1-\frac{\lambda_{f}}{\lambda_{m}})}\right]\times \left[\frac{\left(2-\phi )(2+\frac{\lambda_{f}}{\lambda_{m}})-2\phi (1-\frac{\lambda_{f}}{\lambda_{m}}\right)}{\left(2-\phi )(2+\frac{\lambda_{f}}{\lambda_{m}})+\phi (1-\frac{\lambda_{f}}{\lambda_{m}}\right)}\right]$	(8)
Maxwel-Eucken model [38]	$\lambda_{eff}=\lambda_{m}\frac{2\lambda_{m}+\lambda_{f}+2\phi (\lambda_{f}-\lambda_{m})}{2\lambda_{m}+\lambda_{f}-\phi (\lambda_{f}-\lambda_{m})}$	(9)
Ling model [39]	$\lambda_{eff}=\frac{\lambda_{f}\rho_{composite}\varphi_{EG}}{\rho_{f}}$	(10)

where *λ_f_* and *λ_m_* are the thermal conductivity of the fillers (EG) and the thermal conductivity of the matrix, respectively. As a single matrix with only one dispersed phase is considered by those theoretical models, the mixture of SBS and PEG1500 is taken as the matrix when the mass fraction of EG is less than 4 %, otherwise is taken as the filler [40]. *λ_f_* and *λ_m_* can be obtained by Equation (11), which was proposed by Woodside and Messmer [41].

Similarly, air is considered as a part of the dispersed phase due to its great influence on the thermal conductivity of EG, and *λ_EG_* at 6 MPa is deduced for Equation (11). *φ_EG_* is the mass fraction of EG and *ϕ* is the volume fraction of EG determined by the preparation process of the experimental samples using Equation (12). *C_p_* and *C_f_* are the coefficients determined by the properties of the matrix and the filler. The value of *C_f_* is considered to be large when the conductive chains can be easily formed by fillers, while *C_p_* is affected by the crystallinity and crystal size of the matrix. In this study, *C_p_* and *C_f_* are 1.665 and 0.850, respectively, derived from references [27,42].

(11) $$\begin{array}{l} \left\{\begin{array}{l} \lambda_{m}=(\lambda_{SBS}^{1/2}\phi_{SBS}+\lambda_{PEG}^{1/2}\phi_{PEG}{)}^{2}\\ \lambda_{f}=(\lambda_{EG}^{1/2}\phi_{EG}+\lambda_{air}^{1/2}\phi_{air}{)}^{2} \end{array}\right.\varphi_{EG}<4\%\\ \left\{\begin{array}{l} \lambda_{m}=(\lambda_{EG}^{1/2}\phi_{EG}+\lambda_{air}^{1/2}\phi_{air}{)}^{2}\\ \lambda_{f}=(\lambda_{SBS}^{1/2}\phi_{SBS}+\lambda_{PEG}^{1/2}\phi_{PEG}{)}^{2} \end{array}\right.\varphi_{EG}\geq 4\% \end{array}$$

(12) $$\phi =\frac{m_{EG}/\rho_{EG}}{m_{SBS}/\rho_{SBS}+m_{PEG}/\rho_{PEG}+m_{EG}/\rho_{EG}}$$

where *λ_SBS_* and *λ_PEG_* are the thermal conductivities of SBS, PEG1500, which are measured in Section 3.2 to be 0.180 and 0.346 W/(m·K), respectively. *λ_EG_* as the thermal conductivity of EG is 160.2 W/(m·K) [43], and *λ_air_* as the thermal conductivity of air is 0.026 W/(m·K) [35]. *ϕ_SBS_*, *ϕ_PEG_* and *ϕ_air_* are the volume fractions of SBS, PEG1500 and air, respectively. *ρ_SBS_*, *ρ_PEG_* and *ρ_EG_* are measured to be 781, 1200 and 1353 kg/m^3^ at the pressure of 6 MPa, respectively, *ρ_air_* is 1.29 kg/m^3^ and *ρ_composite_* are shown in Table 1. As the mass fractions of SBS in the samples increase from 0% to 50%, *λ_m_* is considered to gradually decrease from 0.346 to 0.238 W/(m·K), and *λ_f_* is revised to be 67.43 W/(m·K).

**Figure 11:**
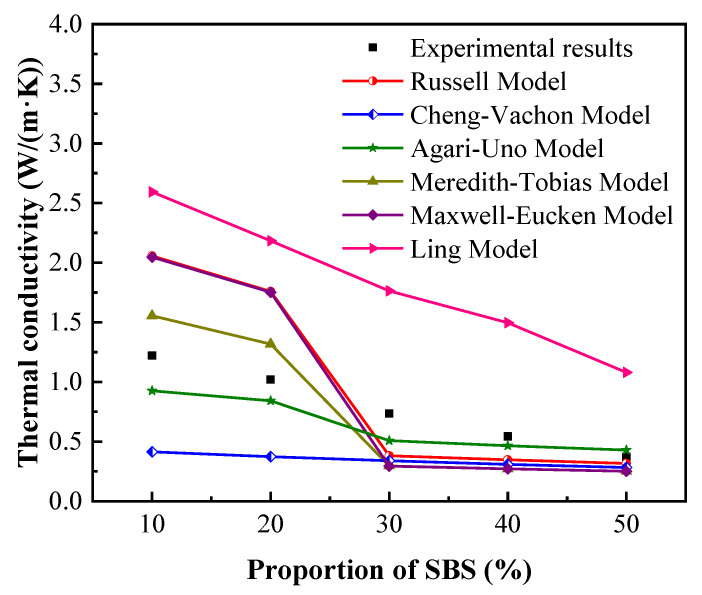
Theoretical and experimental thermal conductivities of composite flexible PCMs at 12.5 °C. The equations of Russell Model, Cheng-Vachon Model, Agari-Uno Model, Meredith-Tobias Model, Maxwell-Eucken Model, Ling Model are from [25,26,27,37,38,39], respectively.

As shown in Figure 11, both the theoretical and experimental thermal conductivities of the composite PCMs with different mass ratios of SBS have the same trend, i.e., decreasing with the increase in the SBS content. It is notable that the variation rate of the experimental results is more apparent with different samples compared to those of Cheng-Vachon model while the prediction values are greatly overestimated by Ling model. In the present study, SBS obviously reduced thermal conductivity of the composite by forming a film on EG. However, the predicted value in the Ling model only depends on *ρ_EG_* and *λ_EG,_* and the thermal conductivityof the matrix is completely ignored, inducing the large estimated values. As for Cheng-Vachon model, EG is fixedly regarded as the dispersed phase regardless of its volume fraction and leads to the low predicted thermal conductivities of the composites.

The theoretical thermal conductivities of Russel model, Agari-Uno model, Meredith-Tobias model and Maxwell-Eucken model slightly deviate from the experimental results for PES50, among which the predicted value of the Agari-Uno model is the closest to the experimental result, overestimating the *λ_eff_* by 15.8%. The deviation sharply increases for low concentration of SBS in samples, and those of theoretical thermal conductivities of PES10 are 68.4%, 24.2%, 27.4% and 67.6% of the experimental results, respectively. Agari-Uno model is considered to be a relatively suitable method to predict the thermal conductivities of such composites in the present study with the average deviation of 20.5%. 

The air inside the composites greatly restricts the heat transfer at the interface between the matrix and nano-filler and increases the thermal resistance, which cannot be neglected in the theoretical prediction of thermal conductivities. For Rusell model, Meredith-Tobias model and Maxwell-Eucken model, the predicted values are not fully match with the experimental results and it is underestimated and overestimated when the concentration of SBS is high and low, respectively. The possible reason is that even the reduction of thermal conductivity caused by the theoretical volume fraction of air is considered in these models, it is difficult to avoid the involvement of air during the process of synthesizing composites as shown in Table 1. The neglected interfacial thermal resistance between particles and matrix leads to the theoretical predicted value being relatively large especially when EG is regarded as the matrix. 

In Agari-Uno model, thermal resistance of interface is considered by *C_f_* and *C_p_*. It is determined by the matrix and filler, which affects the thermal conductivities of the composite PCMs by the effects of heat transfer path and it might be much more obvious when the mass fraction of EG is more than 20%. As the mass fraction of EG is less than 5% in the present study, the prediction is sightly lower than the experimental results and the influence of air is considered by the model, leading a relatively accurate prediction.

## 4. Conclusions

In the present study, a steady-state experimental device was built that could measure the thermal conductivities of samples under different temperatures or phases. Then, the copolymer SBS was added into PEG1500 with 5 wt.% EG as an additive to form a flexible composite PCM. Extensive experiments were conducted to obtain the thermal conductivities of the composite PCMs under various conditions, and the following conclusions can be drawn:

(1) The accuracy of the experimental rig was verified by measuring the thermal conductivity of 304 stainless steel and deionized water. The results showed that the deviation between the thermal conductivity of 304 stainless steel and the literature was within 4%, and that of deionized water was within 8.3% at different temperatures.

(2) The thermal conductivity of SBS itself was 0.18 W/(m·K) and slightly increased with the increase in temperature, rather than the thermal conductivity of composite PCM, which decreased rapidly with the increase in the SBS content. After the integration with 10 wt.% SBS, the thermal conductivity of PES0 dropped sharply to 1.197 from 1.97 W/(m·K), and the decline ratio was 39.2%, which was up to 81.6% when the addition ratio of SBS was 50 wt.%. When the SBS content was constant, the thermal conductivity of all samples increased sightly at first and then decreased with the increase in temperature, and the peak thermal conductivity might appear near the melting phase-change temperature of the composite PCM.

(3) The deviations of the theoretical thermal conductivities predicted by the Russell model, Cheng-Vachon model, Agrai-Uno model, Meredith-Tobias model, Maxwell-Eucken model and Ling model were averagely 47.9%, 49.9%, 20.5%, 39.7%, 56.3% and 146.7% respectively. Agari-Uno model was considered to be the relatively suitable method to predict the thermal conductivities of samples in the present study. In general, the influence of air on the experimental measurement and theoretical prediction of thermal conductivity should be further investigated.

## Figures and Tables

**Table 1:** The components and properties of flexible composite PCMs.

Samples	PEG1500/g	EG/g	SBS/g	Height/mm	Mass/g	Average Density/(kg/m^3^)
PES0	95	5	0	10.24	24.51	1220.10
				32.46	77.9	
				45.84	109.72	
PES10	85.5	4.5	10	15.28	34.62	1156.46
				20.33	46.1	
				46.16	105.19	
PES20	76	4	20	13.84	29.74	1095.35
				31.36	65.21	
				42.38	94.25	
PES30	66.5	3.5	30	15.8	30.41	1010.95
				30.7	61.55	
				45.66	92.48	
PES40	57	3	40	11.2	21.64	1000.93
				30.65	59.3	
				43.71	88.69	
PES50	47.5	2.5	50	13.27	22.48	866.86
				17.41	30.35	
				31.17	52.02	

**Table 2:** Uncertainty analyses of the parameters.

Parameter	Uncertainty Analysis
Measurement parameters	
Diameter, *d* (mm)	0.50 (mm)
Thickness, *h* (mm)	0.02 (mm)
Temperature, *T* (°C)	0.10 (°C)
Export parameters	
$\mathrm{Thickness},\;\frac{\delta h}{h}$ (%)	δh=0.02 (mm) $\frac{\delta h}{h}=\frac{0.02}{45.84}=0.04\%$
$\mathrm{Contact}\;\mathrm{area},\;\frac{\delta A}{A}$ (%)	$A=\frac{\pi }{4}d^{2}$ $\delta A=(0.0505^{2}-0.05^{2})\frac{\pi }{4}=3.95\times 10^{-5}\;(m^{2})$ $\frac{\delta A}{A}=\frac{3.95\times 10^{-5}}{\frac{\pi }{4}\times 0.05^{2}}=2.01\%$
$\mathrm{Difference}\;\mathrm{in}\;\mathrm{temperature},\;\frac{\delta \Delta T}{\Delta T}$ (%)	δΔT=0.10 (°C) $\frac{\delta \Delta T}{\Delta T}=\frac{0.10}{2.53}=3.95\%$
$\mathrm{Heat}\;\mathrm{flow},\;\frac{\delta Q}{Q}$ (%)	$\begin{array}{ll} \delta Q_{1} & =\sqrt{\begin{array}{l} (\lambda_{st}\frac{\Delta t}{\Delta x}\delta A{)}^{2}+(A\frac{\Delta t}{\Delta x}\Delta \lambda_{st}{)}^{2}+\\ (\lambda_{st}\frac{\Delta A}{\Delta x}\delta \Delta t{)}^{2}+(\lambda_{st}A\frac{\Delta t}{{\left(\Delta x\right)}^{2}}\delta \Delta x{)}^{2} \end{array}}\\ & =\sqrt{\begin{array}{l} (10.69\times \frac{2.53}{0.02}\times 0.0000395{)}^{2}+\\ (0.001963\times \frac{2.53}{0.02}\times 0.10{)}^{2}+\\ (10.69\times \frac{0.001963}{0.02}\times 0.10{)}^{2}+\\ (10.69\times 0.001963\times \frac{2.53}{{\left(0.02\right)}^{2}}\times 0.001{)}^{2} \end{array}}\\ & =0.1791\;(W) \end{array}$ $\begin{array}{ll} \frac{\delta Q}{Q} & =\sqrt{(\frac{\delta Q_{1}}{Q_{1}}{)}^{2}+(\frac{\delta Q_{2}}{Q_{2}}{)}^{2}}\\ & =\sqrt{(\frac{0.1791}{2.7034}{)}^{2}+(0.0116{)}^{2}}=6.73\% \end{array}$
$\mathrm{Effective}\;\mathrm{thermal}\;\mathrm{conductivity},\;\frac{\delta \lambda_{e}}{\lambda_{e}}$ (%)	$\begin{array}{ll} \frac{\delta \lambda_{e}}{\lambda_{e}} & =\sqrt{{\left(\frac{\delta Q}{Q}\right)}^{2}+{\left(\frac{\delta \Delta T}{\Delta T}\right)}^{2}+{\left(\frac{\delta h}{h}\right)}^{2}+{\left(\frac{\delta A}{A}\right)}^{2}}\\ & =\sqrt{{\left(0.0673\right)}^{2}+{\left(0.0395\right)}^{2}+{\left(0.0005\right)}^{2}+{\left(0.0201\right)}^{2}}\\ & =8.06\% \end{array}$

## Data Availability

Not applicable.
